# Effects of online and offline trigeminal nerve stimulation on visuomotor learning

**DOI:** 10.3389/fnhum.2024.1436365

**Published:** 2024-10-17

**Authors:** Diego E. Arias, Christopher A. Buneo

**Affiliations:** Visuomotor Learning Lab, School of Biological and Health Systems Engineering, Arizona State University, Tempe, AZ, United States

**Keywords:** trigeminal nerve stimulation, motor learning, visuomotor perturbation, neuromodulation, rehabilitation

## Abstract

**Introduction:**

A current thrust in neurology involves using exogenous neuromodulation of cranial nerves (e.g, vagus, trigeminal) to treat the signs and symptoms of various neurological disorders. These techniques also have the potential to augment cognitive and/or sensorimotor functions in healthy individuals. Although much is known about the clinical effects of trigeminal nerve stimulation (TNS), effects on sensorimotor and cognitive functions such as learning have received less attention, despite their potential impact on neurorehabilitation. Here we describe the results of experiments aimed at assessing the effects of TNS on motor learning, which was behaviorally characterized using an upper extremity visuomotor adaptation paradigm.

**Objective:**

Assessing the effects of TNS on motor learning.

**Methods:**

Motor learning was behaviorally characterized using an upper extremity visuomotor adaptation paradigm. In Experiment 1, effects of offline TNS using clinically tested frequencies (120 and 60 Hz) were characterized. Sixty-three healthy young adults received TNS before performing a task that involved reaching with perturbed hand visual feedback. In Experiment 2, the effects of 120 and 60 Hz online TNS were characterized with the same task. Sixty-three new participants received either TNS or sham stimulation concurrently with perturbed visual feedback.

**Results:**

Experiment 1 results showed that 60 Hz stimulation was associated with slower rates of learning than both sham and 120 Hz stimulation, indicating frequency-dependent effects of TNS. Experiment 2 however showed no significant differences among stimulation groups. A post-hoc, cross-study comparison of the 60 Hz offline and online TNS results showed a statistically significant improvement in learning rates with online stimulation relative to offline, pointing to timing-dependent effects of TNS on visuomotor learning.

**Discussion:**

The results indicate that both the frequency and timing of TNS can influence rates of motor learning in healthy adults. This suggests that optimization of one or both parameters could potentially increase learning rates, which would provide new avenues for enhancing performance in healthy individuals and augmenting rehabilitation in patients with sensorimotor dysfunction resulting from stroke or other neurological disorders.

## 1 Introduction

A large body of evidence supports the idea that neuromodulation can help ameliorate the symptoms of neurological disorders (Johnson et al., 2013) and enhance cognitive and other functions, including learning (Meinzer et al., 2012). Although initial work in this area focused on direct neuromodulation of the brain via transcranial application of electrical or magnetic stimulation, more recent studies have shown that neuroplasticity and learning can also be enhanced by more indirect means, i.e., via electrical stimulation of cranial nerves (Luckey et al., 2023). This approach was initially based on work in animal models showing that vagus nerve stimulation (VNS) paired with delivery of auditory stimuli enhanced neuroplasticity in the auditory cortex (Engineer et al., 2011; Shetake et al., 2012). These findings were expanded to motor areas where it was shown that repeatedly pairing VNS with movement led to similar reorganization of the primary motor cortex (Porter et al., 2012), setting a precedent for recruiting bottom-up mechanisms to modulate motor behavior.

These promising results led to a series of studies in humans that were designed to assess potential clinical applications of VNS, i.e., as treatment for stroke. For instance, a pilot study showed that stroke patients receiving VNS combined with standard rehabilitation exhibited greater scores on the Fugl-Meyer Assessment for upper extremity (FMA-UE) after 6 weeks than patients who received rehabilitation alone (Dawson et al., 2016). A follow-up sham-controlled study demonstrated similar results, including a sustained effect after 90 days compared to the sham group (Kimberley et al., 2018). This was followed by a multi-site, double-blinded, randomized, sham-controlled study of 108 stroke patients which confirmed that VNS paired with standard therapy led to greater functional improvement than therapy alone (Dawson et al., 2021). Subsequently, the FDA approved VNS as a treatment of chronic upper limb dysfunction associated with ischemic stroke.

Although this approval was among the most promising recent developments in stroke rehabilitation, conventional VNS (cVNS) requires a surgically implanted electrode in the left cervical vagus (Wheless et al., 2018), which has raised concerns regarding cost, accessibility, and side effects (Révész et al., 2016; Yap et al., 2020). This has motivated efforts to find methods for stimulating the vagus nerve non-invasively. For example, transcutaneous VNS delivered to the anterior aspect of the neck is currently being investigated as an adjuvant to stroke rehabilitation (Van Der Meij et al., 2020), and is already being used as treatment for other neurological disorders such as migraine and cluster headache (Najib et al., 2022). Others have explored transcutaneous stimulation of the auricular branches of the vagus nerve (taVNS). In one such study, stroke survivors showed an increase in FMA-UE scores after 18 rehabilitation sessions consisting of repetitive task-specific movements paired with taVNS (Redgrave et al., 2018). A similar cohort of stroke patients who received taVNS combined with robotic-assisted therapy also showed improvements in FMA-UE scores compared to sham after 2 weeks of rehabilitation (Capone et al., 2017). Although non-invasive VNS alternatives may be able to circumvent some of the issues associated with cVNS, several lines of evidence indicate that the transcutaneous stimulation of more accessible cranial nerves, such as the trigeminal nerve, might produce similar neuromodulatory effects with few or no side effects.

Trigeminal nerve stimulation (TNS) is a non-invasive neuromodulation technique where low-intensity electric current is delivered to the different branches of this nerve in order to indirectly modulate brain activity. Although the mechanisms underlying TNS effects are not well understood, the rationale behind its application is analogous to that of VNS. That is, both TNS and VNS are thought to result in activation of ascending pathways to brain stem nuclei such as the nucleus tractus solitarius (NTS) and the locus coeruleus (LC), which are known to exert neuromodulatory effects on the cerebral cortex (Sara, 2009). TNS also has a similar therapeutic profile as VNS, with demonstrated beneficial effects on depression, drug-resistant epilepsy (DRE), attention deficit hyperactivity disorder (ADHD), migraine, and other disorders (DeGiorgio et al., 2013; Schoenen et al., 2013; McGough et al., 2019). Other evidence from imaging and neurophysiological studies in animal models and humans supports the idea that TNS modulates brain activity. For instance, a significant reduction in intra-and interhemispheric coherence in the beta band was found in healthy adults receiving TNS, suggesting that it induces neural desynchronization (Ginatempo et al., 2018b), similar to what has been observed in animal studies (Fanselow et al., 2000; Mercante et al., 2017). In another study, DRE patients showed a statistically significant increase in cortical perfusion in limbic and temporal areas after receiving TNS for 20 min (Mercante et al., 2021). In migraine patients, baseline hypometabolism in the orbitofrontal and anterior cingulate cortices was found to be normalized following 3 months of TNS (Magis et al., 2017). The ability of TNS to modulate cortical activity might also explain its effectiveness in reducing markers of physiological stress and improving sleep quality and mood (Tyler, 2017; Tyler et al., 2015), as well as its effects on reaction times, which were found to be significantly faster following single sessions of TNS compared to sham (McIntire et al., 2019).

Although neuroanatomical and neurophysiological data suggest that TNS could have potent effects on cortical networks associated with sensorimotor functions, a notion supported by studies of patients with mild traumatic brain injury, multiple sclerosis, and cerebral palsy (Ignatova et al., 2018; Leonard et al., 2017; Tyler et al., 2019), to our knowledge no studies have directly assessed the effects of TNS on motor *learning* in human subjects. Such experiments could provide critical insights regarding TNS’s potential as an adjuvant to conventional neurorehabilitation. Here we describe two pilot studies designed to assess the effects of single-session TNS on motor learning in healthy adults. We focused on motor learning, which refers to the ability of motor systems to acquire new skills, as its underlying mechanisms are likely relevant for relearning skills that have been impaired due to neurological damage (Krakauer, 2006) or degeneration associated with the normal aging process (Voelcker-Rehage, 2008). In both experiments described here, TNS was delivered to different groups of participants at either 60 or 120 Hz, frequencies which have been shown to be associated with therapeutic effects in ADHD, DRE and migraine (DeGiorgio et al., 2013; Schoenen et al., 2013; McGough et al., 2019), under the hypothesis that one or both would result in more rapid rates of learning. The two studies differed however in the timing of TNS delivery, i.e., online (during task performance) vs. offline (prior to task performance). The use of different timings was motivated by previous studies using transcranial neuromodulation approaches, which showed that in single-session experiments, online stimulation appeared to have more pronounced effects than offline stimulation (Martirosov et al., 2022). As a result, we sought to determine if a similar phenomenon applies to TNS. To that end, in Experiment 1, TNS was delivered offline while in Experiment 2, TNS was delivered online.

## 2 Materials and methods

### 2.1 Subjects

One-hundred and thirty-two (132) right-handed individuals without prior history of neurological or psychiatric disorders participated in these experiments. To determine the appropriate sample size for each independent group, we conducted a power analysis in R Statistical Software (v4.1.0; R Core Team, 2021) More specifically, assuming a large effect size (*f* = 0.4), a significance level of 0.05, a desired power of 0.8, *k* = 3 groups, and balanced, one-way analyses of variance, the number of subjects in each group was determined to be 21.

For Experiment 1, 67 participants were recruited. Data sets from four participants were discarded: one participant withdrew in the middle of the experiment due to an unforeseen conflict in her schedule, while the other three participants were excluded due to noisy kinematic data on more than 10% of trials (See Data Analysis). Data from the remaining 63 participants (18–36 y/o; 22.75 ± 4.6 y/o; 32 female and 31 men) were analyzed. For Experiment 2, 65 participants were recruited. Data sets from two participants were discarded: one due to complaints about eye strain which resulted in abandonment of the experiment and the other due to a deviation from the protocol that was discovered *post hoc*. Thus, data from 63 participants (18–32 y/o; 23.2 ± 3.9 y/o; 12 female and 51 male) were analyzed. Demographic information for each group in both experiments can be found in the Supplementary Tables 1, 2.

Written consent was obtained from all participants in accordance with the Declaration of Helsinki. All subjects declared themselves as right-handed, but their handedness was also assessed using the Edinburgh Handedness Inventory (short form) (Veale, 2014). All subjects reported having normal or corrected-to-normal vision. Eligible individuals participated in only one experiment and the Arizona State University Institutional Review Board approved both experimental protocols.

Both studies were designed as single-blind, randomized, sham-controlled experiments. Block randomization was used to ensure equal sample size across groups using Sealed Enveloped.^1^ In both experiments, subjects were assigned to one of three different groups defined by the frequency of the stimulation: 120 Hz, 60 Hz, and sham.

### 2.2 Apparatus

**Figure 1:**
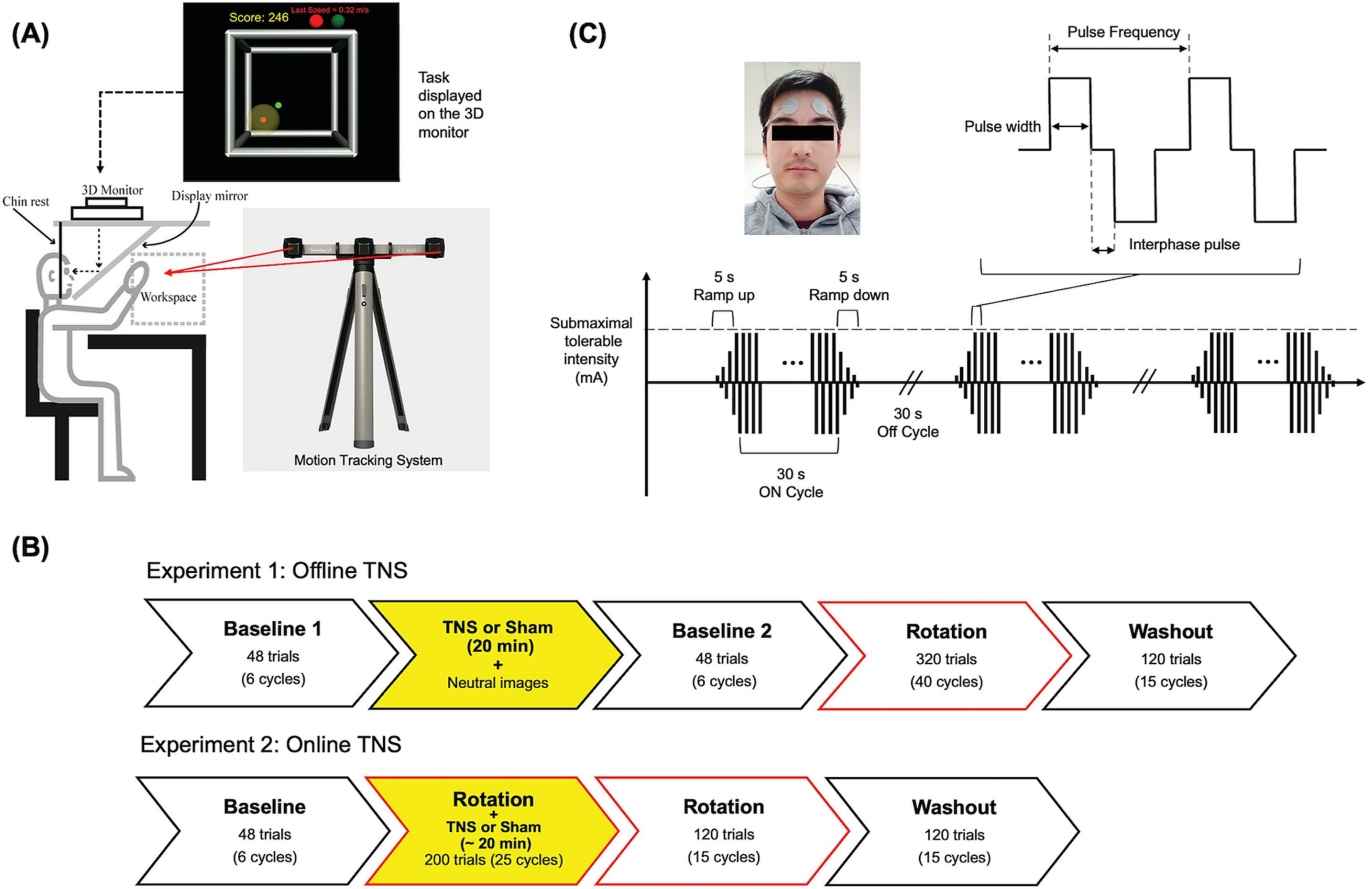
Experimental apparatus **(A)**, experimental designs **(B)**, and stimulation protocol **(C)**. Adapted from Arias and Buneo (2022).

Participants performed visually guided reaching movements within a semi-immersive 3D virtual reality (VR) environment. An active LED motion tracking system (Visualeyez VZ-3000; Phoenix Technologies, Inc.) was used to track arm movements. Visual images rendered in Vizard 3.0 (WorldViz Inc.) were displayed on a stereoscopic 3D monitor (Dimension Technologies Inc.) and projected onto a mirror that was embedded within a metal plate oriented at a 45° angle with respect to the monitor. During the experiment, participants were seated with their head positioned on a chin rest and with their eyes aligned with the center of the mirror. To prevent the arm from being directly viewed, movements were performed behind the metal plate, but visual feedback of the fingertip was provided to the participants in the form of a virtual cursor that was projected onto the mirror. Figure 1A shows the described setup.

### 2.3 Behavioral task

Participants performed an upper extremity visuomotor rotation task, which has been commonly used to investigate motor adaptation, a component of motor learning (Della-Maggiore et al., 2015). This task engages processes involved in both low-level motor execution and high level of cognition and has previously been used to explore the effects of other neuromodulation techniques such as transcranial direct current stimulation (tDCS) and transcranial magnetic stimulation on motor learning (Galea et al., 2011; Herzfeld et al., 2014; Koch et al., 2020). The task required participants to gradually adapt to an imposed discrepancy between hand motion and corresponding visual feedback (Krakauer and Mazzoni, 2011). To this end, center-out reaching movements were made to eight targets located 10 cm away from the starting position and 45° apart within a vertical plane. Subjects were instructed to move as fast and as accurately as possible, and they received online visual feedback of their movements as well as terminal auditory feedback for correctly hitting the target. To ensure that participants used approximately the same range of movement velocities, they also received terminal visual feedback about their peak movement velocity and were encouraged to achieve PVs greater than or equal to 0.5 m/s. Feedback about movement accuracy was also provided using a point-based scoring system, which was designed to make the task more engaging. In addition, a message encouraging subjects to take a break was displayed every 25 trials to reduce the fatigue in the arm though subjects could also rest as needed.

Experiment 1 – Offline Stimulation (Figure 1B Top): Participants began with a familiarization block, where they were instructed on how to perform the task with veridical visual feedback and no stimulation. This was followed by one experimental baseline block of 48 trials, also performed with veridical visual feedback and no stimulation (‘baseline 1’). After baseline 1, participants received either 120 Hz-TNS, 60 Hz-TNS or sham-TNS for 20 min under resting conditions. During this period, they viewed images of neutral valence and arousal obtained from the International Affective Picture System (IAPS; 131 images with mean valence scores: 5.17 ± 0.4, mean arousal scores: 3.36 ± 0.76). This was done in order to minimize variations in emotional states across participants (Lang et al., 2008). Immediately after the stimulation block, a second baseline block of 48 trials with veridical feedback and no stimulation was performed (‘baseline 2’) to determine whether the stimulation affected baseline motor performance. After baseline 2, a single rotation block of 320 trials involving a 30° counterclockwise (CCW) rotation of hand visual feedback performed without stimulation was performed. The perturbation was applied only in the vertical plane containing the targets even though participants were able to move freely within a 3D space. The experiment ended with a washout block of 120 trials, where the rotation was removed, and no stimulation was applied.

Experiment 2 – Online Stimulation (Figure 1B Bottom): Like Experiment 1, Experiment 2 began with a familiarization block followed by a baseline block of 48 trials to assess participant performance. Following this, participants experienced a 30° CCW rotation of the hand visual feedback and concurrent TNS for 200 trials. After “the TNS + rotation” block was completed, participants performed another 120 trials with rotated feedback but no stimulation. The experiment ended with a washout block of 120 trials, where the rotation was removed, and no stimulation was applied.

In both experiments, participants were given no prior knowledge regarding the introduction and removal of the rotated visual feedback and no instructions regarding strategies to counteract the rotation. Also, immediately after either receiving TNS or sham stimulation, subjects were asked to mark on a diagram of the face anatomy whether they had felt the stimulation and the location of any sensations perceived. Finally, once the experiment was completed, subjects filled out a survey to determine potential side effects regarding the stimulation as well as their perceived level of attention during the task.

### 2.4 Stimulation protocol

TNS was applied using two round, 3.2 cm diameter, surface electrodes (Axelgaard Manufacturing Co., Ltd.) placed on either side of the forehead to bilaterally stimulate the supraorbital branches of the trigeminal nerve (DeGiorgio et al., 2006). Before the electrodes’ placement, the forehead was cleaned with an alcohol prep pad to reduce the impedance between the electrode and the skin. Figure 1C shows the stimulation protocol. A biphasic symmetric square waveform was delivered using a peripheral nerve stimulator approved for human research (DS8R, Digitimer Ltd.). The stimulator was triggered using a function generator (AFG 3022B, Tektronix Ltd.), controlled by a custom graphical user interphase written in MATLAB (The MathWorks, Inc.) that also allowed setting stimulation parameters such as the frequency, current amplitude, and pulse width. TNS was delivered in cycles of 30 s ON and 30 s OFF similar to previous studies (DeGiorgio et al., 2006; Ginatempo et al., 2018b; McGough et al., 2019). During the ON periods, the current was ramped up/down over 5 s to make the ON/OFF transitions more comfortable. Other parameters such as the pulse width (250 μs) and interphase interval (1 μs) were the same for both groups.

For Experiment 1, TNS was delivered prior to task performance (offline) for 20 min. Therefore, due to the ON/OFF periods, participants received 10 min of effective TNS. On the other hand, for Experiment 2, TNS was delivered online, i.e., concurrent with task performance, and the stimulation time varied depending on how long subjects took to perform the task. For this reason, TNS was delivered only during the first 200 trials (25 cycles) of the rotation block, which were completed in ~20 min on average. This way, the stimulation doses received in both experiments were comparable.

Before the stimulation session began, participants were told that a comfortable level of stimulation would be selected and that they may or may not feel the stimulation. The experimenter then gradually increased the stimulation current until it reached a tolerable level that was greater than 0 mA but less than the established maximum safety level of 6 mA. Once the intensity level was determined, it was used throughout the experiment, but participants could still request to reduce or stop the stimulation at any time. The intensity setting procedure was also applied to sham participants even though no stimulation was provided. Thus, electrodes were placed in the same location as the experimental groups and the stimulator still generated the same sounds as when it was delivering current, which helped to reinforce the sham. In both experiments, immediately after the stimulation session was completed (either active or sham) subjects were asked whether they had felt the stimulation as well as the location of any sensations.

### 2.5 Data analysis

**Figure 2:**
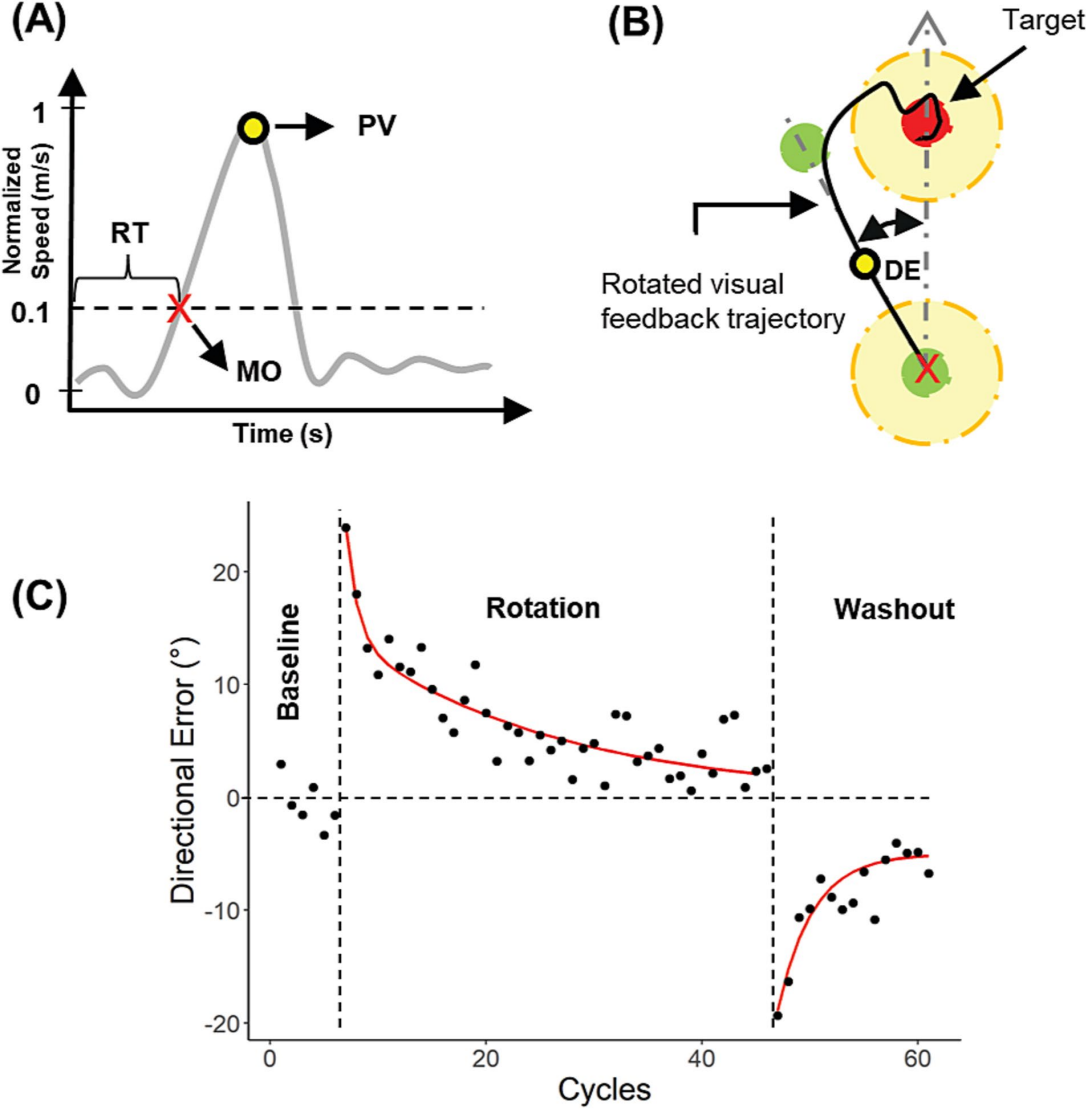
Calculation and analysis of directional errors (DE). **(A)** Calculation of movement onset (MO), reaction time (RT) and peak velocity (PV) from a velocity profile. **(B)** DE calculation. **(C)** DEs for a single subject plotted against cycles for all experimental blocks in Exp. 2.

Kinematic analyses were performed in MATLAB. Hand movements were sampled at 125 Hz, filtered using a low-pass 2nd order Butterworth filter with a cutoff frequency of 6.25 Hz, and differentiated to obtain hand movement velocities. Velocity profiles (Figure 2A) were used to obtain movement and behavioral parameters such as movement onset (MO), peak velocity (PV), and reaction time (RT). MO was estimated as the first point in time at which the movement exceeded 10% of the PV. RT was measured from the moment the visual target was presented until MO. To determine PV, RT, and MO, a semi-automatic script written in MATLAB was used. Each trial was reviewed, and if MO was incorrectly detected, it was adjusted manually. Additionally, if a velocity profile exhibited multiple peaks or the movement trajectory did not follow an initially straight path, the trial was deleted.

Visuomotor performance was quantified by the directional error (DE), defined as the angular difference between the hand position at PV and the target direction (Figure 2B). Individual DEs during the rotation and washout blocks were corrected by removing any intrinsic directional biases observed during baseline blocks (Ghilardi et al., 1995). More specifically, we first calculated the mean directional error for each target direction during each baseline block. For Experiment 1, we used data from baseline 1 (pre-stimulation) instead of baseline 2 (post-stimulation) to estimate the intrinsic bias, in case the latter was affected in some way by the stimulation. Subsequently, these biases were subtracted from the directional error on each trial of the rotation and washout blocks.

Cycles were defined as eight consecutive trials (one per target direction). Within the cycles, any trial DE, PV, or RT exceeding two standard deviations from the mean was deleted. Subsequent analyses were performed on the binned data from the remaining trials. Overall, datasets with more than 10% of the total movements deleted were not analyzed further.

Statistical analyses were performed in R Statistical Software. Following the convention of several previous studies (Krakauer et al., 2005, 2000, 1999), participant’s mean DEs during the rotation block were fitted to a double exponential model (see Equation 1). This allowed for quantification of the fast and slow learning processes that drive motor adaptation. To this end, a nonlinear least squares procedure based on the Levenberg–Marquardt algorithm was used (nlsLM function in R) to fit the following model:


(1) $$\hat{DE_{r}}=C_{1}e^{-\alpha i}+C_{2}e^{-\beta i}$$

where $\hat{DE_{r}}$ is the estimated directional error during the rotation block, *α* and *β* represent the fast and slow learning rates, *C_1_* and *C_2_* are the magnitudes of each exponential and *i* is the *i*-th cycle during the rotation block. We assumed that α and β > 0, α > β and *C_1_* and *C_2_* > 0.

On the other hand, mean DEs during the washout block were fitted to a single exponential model (Equation 2):


(2) $$\hat{DE_{w}}=Ae^{-\gamma i}+C$$

where $\hat{DE_{w}}$ is the estimated directional error during the washout block, *γ* represents the forgetting rate, *A* is the magnitude of the exponential, *i* is *i*-th cycle during the washout block and *C* is a constant. We assumed that *A* < 0 (error in the opposite direction) and γ > 0. Example DEs and exponential curve fits are shown for a representative subject in Figure 2C.

Analyses of baseline performance: For Experiment 1, two-factor mixed ANOVAs were conducted to examine the effects of the stimulation groups (between-subjects factor: stim group) and pre/post stimulation (within-subjects factor: epoch) on motor performance (mean RT, PV, and DE across cycles between baselines 1 and 2). For Experiment 2, one-way ANOVAs were conducted to compare RT, PV, and DE between stimulation groups during the baseline block. For both experiments, Shapiro–Wilk tests were performed prior to the ANOVA tests to confirm normality.

Analyses of adaptation and post-adaptation performance: In both experiments, learning rate coefficients were not normally distributed, as assessed by the Shapiro–Wilk test and via examination of QQ plots. As a result, to compare the learning and forgetting rates across different groups, the non-parametric Kruskal-Wallis test was used. Pairwise-multiple comparisons using Dunn’s test were performed if significant results were found and *p*-values were adjusted (*p*_adj_) using the Benjamin-Hochberg correction.

Changes in RT, PV, and DE were also compared between the early and late phases of the adaptation block. To estimate early adaptation performance, the average RT, PV, and DE across the first 15 cycles were calculated for each subject’s data set. Similarly, to estimate late adaptation performance, the mean values across the last 15 cycles were computed. Comparisons were performed using two-factor mixed ANOVAs (between-subject factor: stim group; within-subject factor: epoch). A similar approach was used for the de-adaptation performance. The early phase of the de-adaptation was estimated using the average of the first six cycles, while the late phase was estimated based on the mean of the last six cycles. Two-factor mixed ANOVAs were used on the averaged data (between-subjects factor: stim group; within-subjects factor: epoch). If significant results were found, *p*-values were adjusted using the Bonferroni correction.

Finally, cross study comparisons between learning curves and learning rates obtained in Experiments 1 and 2 were performed using the non-parametric Wilcoxon rank-sum test. For all statistical tests, effect sizes were reported for statistically significant results. Different measures of effect size were used depending on the test. For t-tests, Cohen’s d was used. For the ANOVAs, generalized eta squared ( $\eta_{G}^{2}$ ) was used and for the Kruskal-Wallis tests eta squared based on the H-statistic $\eta^{2}\left(H\right)$ was used. Lastly, for the Wilcoxon rank-sum test, r was used, which was obtained be dividing the z statistic by the square root of the sample size.

## 3 Results

### 3.1 Experiment 1: offline stimulation

Average stimulation intensities were similar between active stimulation groups but varied somewhat between male and female participants. On average, participants from the 120 Hz and 60 Hz groups received 3.1 ± 0.7 mA and 3.4 ± 0.8 mA of TNS, respectively. No statistically significant differences in tolerated stimulation intensities between groups were found (t-test, *t*(39.68) = −1.24, *p* = 0.22). Average intensities for male participants (*n* = 18, 3.7 ± 0.7 mA) were generally higher than those of female participants (*n* = 24, 2.9 ± 0.7 mA; t-test, *t*(37.7) = −3.6, *p* < 0.001, Cohen’s *d* = −1.09). Since effects of intensity on learning rates or reported attention levels were generally equivocal (see Other Effects of TNS), these differences were not examined further.

#### 3.1.1 Directional error (DE)

DEs were generally small during the baseline blocks and did not appear to be influenced by TNS delivery. During baseline 1, DEs were close to 0° for all groups (120 Hz: 0.1° ± 1.3, 60 Hz: −0.09° ±1.3, Sham: −0.1° ±1.3) and during baseline 2, DEs were also negligible (120 Hz: 0.3° ± 1.6, 60 Hz: −0.09° ± 1.6, Sham: −0.1° ±1.3). A two-factor mixed ANOVA was used to assess the effects of group and pre/post-stimulation on baseline DEs. This analysis showed no statistically significant effects of group (*F*(2, 60) = 0.36, *p* = 0.697) or pre/post stimulation (*F*(1, 60) = 0.29, *p* = 0.595) on DE. Also, no statistically significant interaction between group and epoch (pre/post-stimulation) was found (*F*(2, 60) = 0.08, *p* = 0.926).

**Figure 3:**
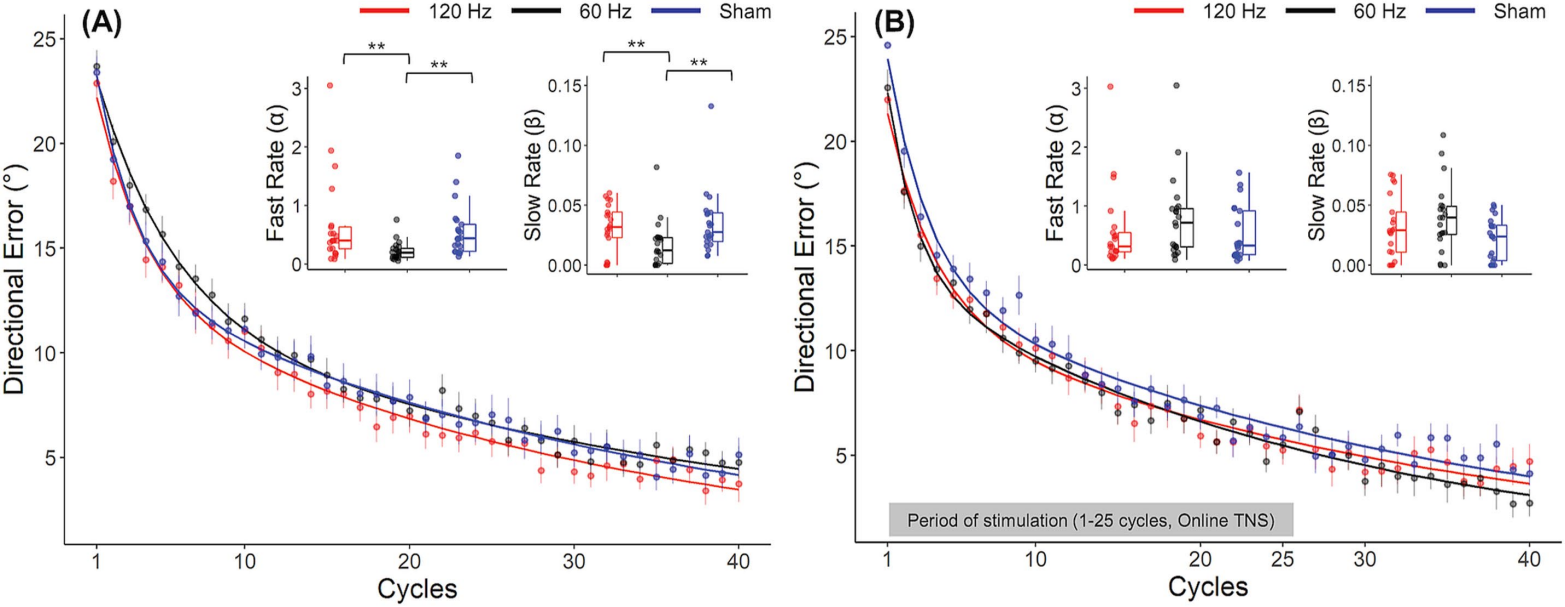
Frequency dependent effects of TNS on rates of motor adaptation. Learning curves for the 120 Hz (red), 60 Hz (black) and sham (blue) for Experiment 1 **(A)** and Experiment 2 **(B)**. Insets: Fast and slow rates obtained from a double exponential model fitted to the average DE data for each group. Error bars represent the SEM. *p ≤ 0.05; **p ≤ 0.01; ***p ≤ 0.001.

Analyses of DEs showed evidence of frequency dependent effects of TNS during the rotation block. DEs for all groups throughout the rotation block are illustrated in the form of learning curves in Figure 3A. As expected, DEs decayed exponentially from approximately 25° at the beginning of the block, (which was close to the perturbation angle of 30°) to nearly 0° at the end, indicating that participants gradually adapted to the rotated environment. A two-factor mixed ANOVA conducted on the DEs obtained during the early and late phases of this block confirmed this decay (Table 1) but failed to capture the more subtle differences that could be observed between groups. As a result, we also analyzed the learning rate coefficients that were obtained by fitting the DEs from the entire block to double exponential models. Visual inspection of these curves in Figure 3A suggests that the 60 Hz group exhibited a slower rate of adaptation than the sham and 120 Hz groups during the early adaptation phase (cycles 1–15). On the other hand, learning curves were initially similar between the 120 Hz and sham groups but gradually diverged, with the 120 Hz group exhibiting slightly smaller DEs than the sham and 60 Hz groups in the late adaptation block (cycles 35–40). Analysis of the corresponding learning rates (Figure 3A) revealed significant differences among groups for both fast and slow rates (Kruskal-Wallis test; *α*: χ^2^(2) = 14.6, *p* < 0.001, $\eta^{2}$ =0.21; *β*: χ^2^(2) = 10.3, *p* = 0.00575, $\eta^{2}$ =0.139). *Post hoc* pairwise comparisons using the Dunn test revealed that the fast and slow rates from the 60 Hz group were significantly different from those of the 120 Hz (α: *p_adj_* = 0.00284, β: *p_adj_* = 0.00977) and sham groups (α: *p_adj_* = 0.00148, β: *p_adj_* = 0.00977). However, the fast and slow rates comparison showed no statistically significant differences between 120 Hz and Sham (α: *p* = 0.705, β: *p* = 0.906).

**Table 1:** Results of two-factor mixed ANOVAs for RT, PV, and DE in Experiment 1.

			Reaction time (RT)			Peak velocity (PV)			Directional error (DE)		
		df	F	*p*	η^𝟐^_G_	F	*p*	η^𝟐^_G_	F	*p*	η^𝟐^_G_
Baselines (b1 and b2)	Groups	(2, 60)	0.21	0.810	0.006	0.80	0.452	0.024	0.36	0.697	0.009
	pre/post	(1, 60)	3.82	0.055	0.006	**21.99**	***p* < 0.001**	**0.023**	0.29	0.595	0.001
	Interaction	(2, 60)	0.36	0.701	0.001	1.59	0.213	0.003	0.08	0.926	<0.001
Rotation	Groups	(2, 60)	0.61	0.545	0.019	0.78	0.463	0.024	0.65	0.524	0.019
	Early/late	(1, 60)	3.41	0.070	0.003	**39.57**	***p* < 0.001**	**0.039**	**1365.86**	***p* < 0.001**	**0.664**
	Interaction	(2, 60)	0.34	0.716	<0.001	0.92	0.405	0.002	0.99	0.378	0.003
Washout	Groups	(2, 60)	0.85	0.432	0.027	1.15	0.322	0.036	0.54	0.583	0.015
	Early/late	(1, 60)	0.28	0.602	<0.001	**13.54**	***p* < 0.001**	**0.008**	**727.96**	***p* < 0.001**	**0.685**
	Interaction	(2, 60)	0.75	0.476	<0.001	0.02	0.981	< 0.001	0.92	0.402	0.005

Bolds values highlight statistically significant results.

No frequency dependent effects of TNS were found during the washout block. During this block, all groups experienced aftereffects, i.e., DEs in the opposite direction that decayed with time, and which is thought to reflect a ‘forgetting’ of the perturbation. As in the rotation block, a two-factor mixed ANOVA conducted on the DEs obtained during the early and late phases confirmed this decay (Table 1). However, learning rate coefficients were highly variable across subjects during this block and as a result the rates at which the DEs decayed did not vary across groups (Kruskal-Wallis test, χ^2^(2) = 4.55, *p* = 0.1).

#### 3.1.2 Reaction time (RT) and peak velocity (PV)

Statistical analyses did not point to strong effects of offline TNS on RT and PV. Table 1 reports results from two-factor mixed ANOVAs conducted on the mean RTs and PVs obtained in the different analysis epochs. Mean RTs for all groups fluctuated between 320 and 360 ms and did not appear to increase or decrease within or across blocks (see Supplementary Table 3). This was confirmed by the ANOVA which showed no statistically significant main effects of stimulation group or epoch on RT in the baseline (pre/post), rotation (early/late), or washout (early/late) blocks. In addition, no statistically significant interaction effects between group and epoch were found.

Mean PVs for all groups fluctuated between 0.45 and 0.6 m/s throughout the experiment (Supplementary Table 3). Despite the absence of clear differences between groups, there was a notable trend within each group, showing a progressive increase from the beginning to the end of each block. For instance, PV increased significantly from the pre- to post-stimulation baseline (*F*(1, 60) = 21.99, *p* < 0.001, $\eta_{G}^{2}$ =0.023), with *post hoc* comparisons indicating this effect was attributed to both the 60 Hz (*p_adj_* = 0.015) and sham (*p_adj_* = 0.015) groups. During the early adaptation phase, all groups experienced a reduction in PV compared to baseline levels, which coincided with the introduction of the perturbation. However, PV increased again toward the latter phase of the adaptation (*F*(2,60) = 39.57, *p* < 0.001, $\eta_{G}^{2}$ =0.039). *Post hoc* multiple comparisons identified significant differences within all groups between early and late adaptation (120 Hz: *p_adj_* = 0.006; 60 Hz: *p_adj_* < 0.001; sham: *p_adj_* = 0.024). Finally, during the washout block, there was a significant increase in PV from the early phase to the late phase (*F*(1, 60) = 13.54, *p* < 0.001, $\eta_{G}^{2}$ =0.08). Pairwise comparisons indicated that this effect was pronounced only within the sham group (*p* = 0.026, *p*_adj_ = 0.078). Here again, however, no statistically significant interactions between stimulation groups and epoch were found. The absence of significant interaction effects between groups and epoch on RT and PV suggests that active TNS had little to no effect on these behavioral and kinematic variables.

### 3.2 Experiment 2: online stimulation

In Experiment 2, average stimulation intensities were similar between active stimulation groups and between genders. Participants in the stimulation groups self-selected similar current levels (60 Hz: 3.3 ± 0.8 mA; 120 Hz: 3.1 ± 0.8 mA). A t-test showed that there was no statistically significant difference between these currents (*t*(40) = −0.67, *p* = 0.51). In contrast to Experiment 1 however, there was no statistically significant difference (t-test, *t*(16.45) = −0.35, *p* = 0.73) in the intensity levels tolerated by male subjects (*n* = 34, 3.2 ± 0.8 mA) and female subjects (*n* = 8, 3.1 ± 0.5 mA).

In contrast to Experiment 1, where the stimulation time was fixed at 20 min, in Experiment 2, the stimulation time depended on how long participants took to complete the first 25 cycles. Despite that, the average duration of the stimulation sessions was approximately 20 min (120 Hz: 21 ± 4.2 min, 60 Hz: 22 ± 4.1 min, sham: 21.8 ± 3 min), similar to Experiment 1. This similarity in stimulation duration facilitated comparisons between the two experiments, as described below.

#### 3.2.1 Directional error (DE)

In contrast to offline TNS, no evidence of frequency dependent effects of online TNS on DE was found. All groups showed average DEs that were close to zero during the baseline block (120 Hz: −0.25° ± 1.1; 60 Hz: 0.48° ± 1.57; Sham: 0.09° ± 1) and a one-way ANOVA showed no statistically significant differences in baseline DEs among groups during this block (*F*(2, 60) = 1.31, *p* = 0.277). Visual inspection of Figure 3B indicates that DEs decreased dramatically between the early and late phases of the rotation block, a difference that was confirmed statistically (Table 2). Figure 2B also shows that during the rotation block, learning rates for the stimulation groups appeared to be slightly faster than those of the sham group. However, no statistically significant differences were found among groups for either the fast rate (Kruskal-Wallis test; χ^2^(2) = 2.76, *p* = 0.25) or slow rate coefficients (Kruskal-Wallis test; χ^2^(2) = 4.46, *p* = 0.11). For the washout block, DEs followed the characteristic reduction in DEs over blocks but no difference in the rates at which performance returned to baseline levels among groups was found (Kruskal-Wallis test; χ^2^(2) = 2.86, *p* = 0.24).

**Table 2:** Results of one-way (baseline block) and two-factor mixed ANOVAs (rotation and washout blocks) for RT, PV, and DE in Experiment 2.

			Reaction time (RT)			Peak velocity (PV)			Directional error (DE)		
		df	F	*p*	η^𝟐^_G_	F	*p*	η^𝟐^_G_	F	*p*	η^𝟐^_G_
Baseline	Groups	(2, 60)	1.17	0.316	0.038	2.72	0.074	0.083	1.31	0.277	0.042
Rotation	Groups	(2, 60)	0.86	0.427	0.026	0.77	0.465	0.021	1.57	0.217	0.040
	Early/late	(1, 60)	**6.62**	**0.013**	**0.006**	**34.04**	***p* < 0.001**	**0.095**	**684.78**	***p* < 0.001**	**0.704**
	Interaction	(2, 60)	2.81	0.068	0.005	0.55	0.581	0.003	0.76	0.473	0.005
Washout	Groups	(2, 60)	1.07	0.349	0.033	0.73	0.488	0.023	0.93	0.399	0.024
	Early/late	(1, 60)	0.86	0.357	<0.001	**13.24**	***p* < 0.001**	**0.010**	**570.34**	***p* < 0.001**	**0.661**
	Interaction	(2, 60)	0.14	0.868	<0.001	0.43	0.653	<0.001	0.37	0.695	0.002

Bolds values highlight statistically significant results.

#### 3.2.2 Reaction time (RT) and peak velocity (PV)

Statistical analyses did not reveal strong effects of online TNS on RT and PV. Table 2 reports the results of these analyses. A one-way ANOVA was used to compare RTs and PVs across groups during the baseline block and a two-factor mixed ANOVA was used to analyze performance during the rotation and washout blocks. Mean RTs fluctuated between 320 and 360 ms, similar to Experiment 1 (Supplementary Table 4). During the rotation block, a significant reduction in RT was noted as the experiment progressed, reflected by a significant main effect of epoch (*F*(1, 60) = 6.62, *p* = 0.013, $\eta_{G}^{2}$ =0.006). *Post hoc* multiple comparisons attributed this effect to the sham group (*p_adj_* < 0.001). No other statistically significant main effects of stimulation group or epoch nor any significant interaction effects were found for any of the blocks.

Mean PVs fluctuated between 0.45 and 0.65 m/s (Supplementary Table 4). No statistically significant main effects of stimulation group were found during baseline. During the rotation block, all groups increased their PV as the study progressed. A statistically significant main effect of epoch (early/late) on PV was found for this block (*F*(1, 60) = 34.04, *p* < 0.001, $\eta_{G}^{2}$ =0.095) and *post hoc* testing revealed that the effect was evident in the 120 Hz and 60 Hz groups (120 Hz: *p* = 0.001, *p_adj_* = 0.003; 60 Hz: *p* = 0.002, *p_adj_* = 0.006; sham: *p* = 0.017, *p_adj_* = 0.051). During the washout, there was also a statistically significant main effect of epoch (*F*(1, 60) = 13.24, *p* < 0.001, $\eta_{G}^{2}$ =0.010) with *post hoc* comparisons indicating no statistically significant differences between the early and late phases for the sham group (*p* = 0.023, *p_adj_* = 0.069) or the 60 Hz (*p* = 0.111, *p_adj_* = 0.333) and 120 Hz (*p* = 0.047, *p_adj_* = 0.141) groups. Again, similar to Experiment 1, the absence of significant interaction effects in this experiment suggests that online TNS had little to no effect on PV and RT.

### 3.3 Cross study comparison

**Figure 4:**
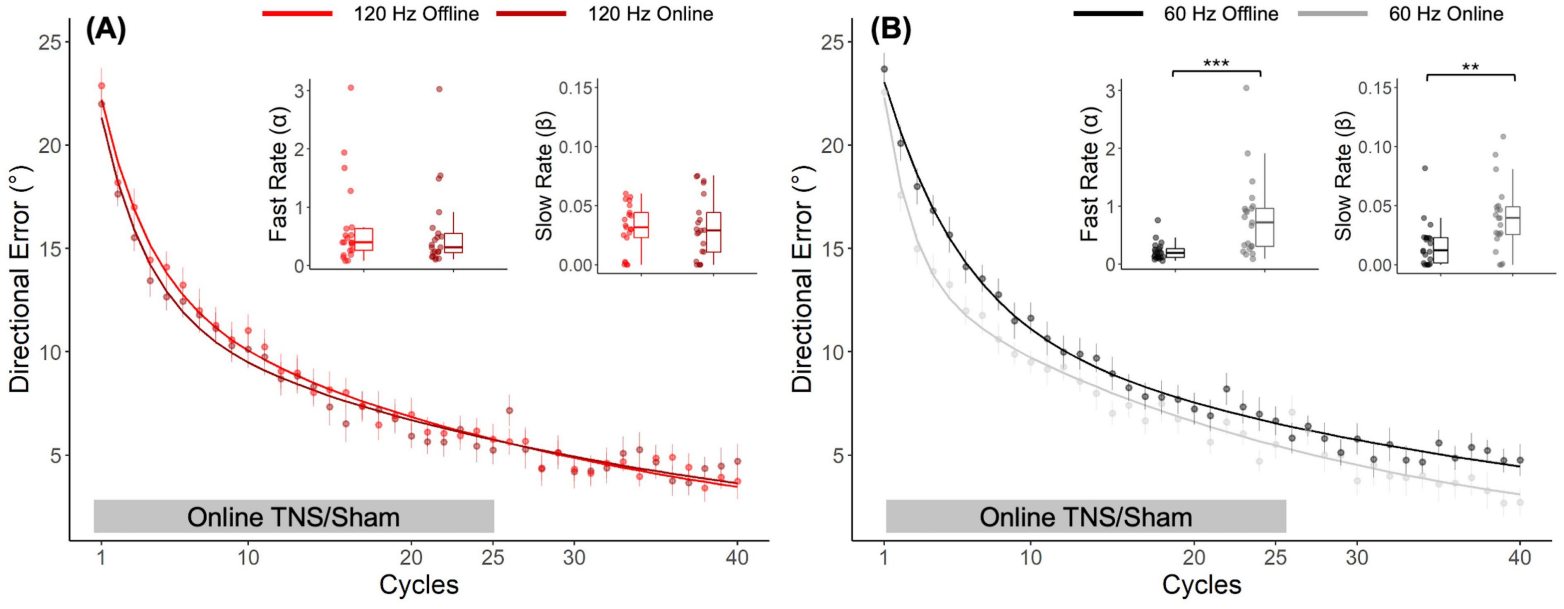
*Post hoc*, cross-study comparison of learning curves from Experiments 1 and 2 points to timing dependent effects of TNS on motor adaptation. Learning curves fitted to the DE for the 120 Hz group **(A)** applied online (maroon bold line) and offline (red bold line). Learning curves fitted to the DE for the 60 Hz group **(B)** applied online (gray bold line) and offline (black bold line). Error bars represent the SEM. *p ≤ 0.05; **p ≤ 0.01; ***p ≤ 0.001.

A post-hoc direct comparison of DEs obtained with offline and online TNS emphasized the importance of stimulation timing on performance. Figure 4A shows the learning curves obtained from the different cohorts of subjects who received 120 Hz TNS in either online (maroon-bold line) or offline mode (red-bold line). As suggested by the largely overlapped learning curves in this figure, learning rates with online 120 Hz were similar to those observed with offline 120 Hz TNS, a result that was confirmed statistically (Wilcoxon rank-sum test, *α*: W = 246, *p* = 0.53; *β*: W = 231, *p* = 0.8). On the other hand, the learning curves shown in Figure 4B suggest that 60 Hz online TNS resulted in markedly faster learning than what was observed with 60 Hz-offline TNS. This observation was confirmed by a statistical analysis that directly compared the learning rates (Wilcoxon rank-sum test: α: W = 71, *p* < 0.001, *r* = 0.58; β: W = 101, *p* = 0.0027, *r* = 0.465).

### 3.4 Sham condition

**Figure 5:**
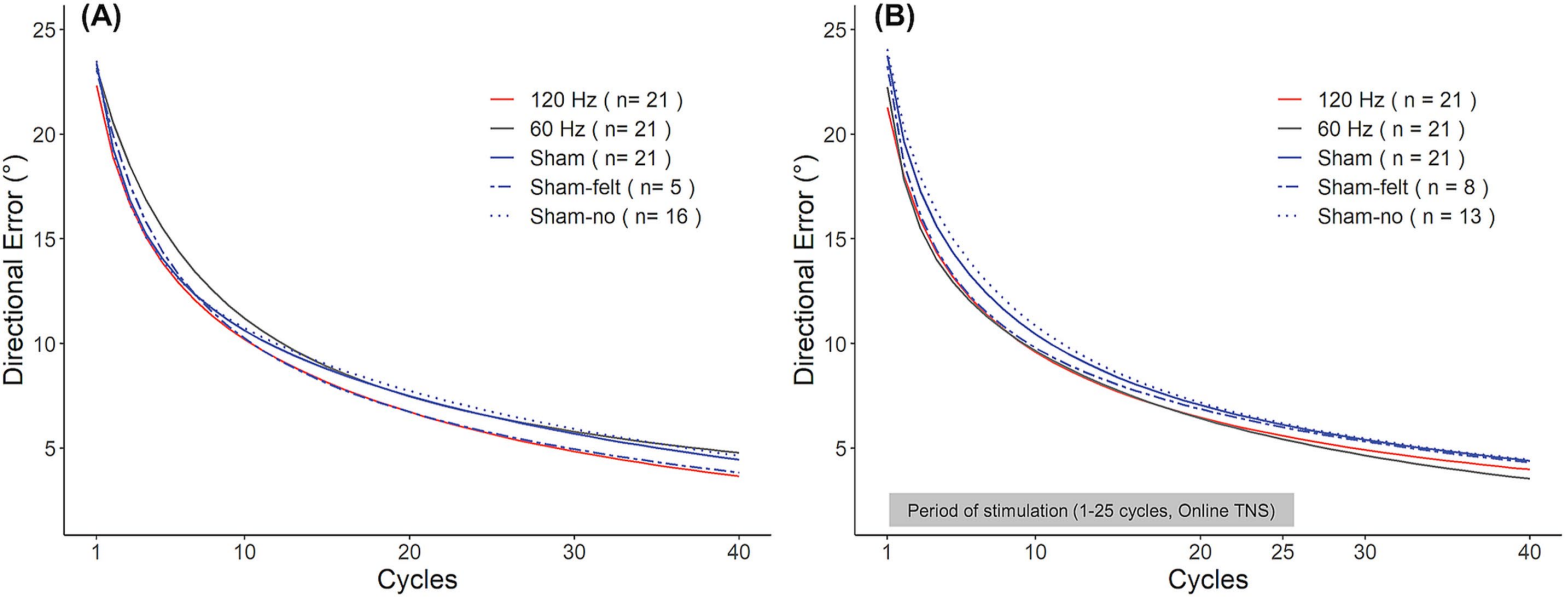
Sham group learning curves for Experiment 1 **(A)** and Experiment 2 **(B)**. Participants in the sham group were split into those who experienced the stimulation (Sham-felt, dashed blue line) and who did not (Sham-no, dotted blue line).

Interestingly, sham subjects appeared to perform differently depending on whether they believed they had received stimulation or not. In Experiment 1, five out of 21 subjects in the sham group reported having perceived the stimulation even though no current was applied to participants in this group. In Experiment 2, this occurred in eight out of 21 subjects. As a result, we explored how the perception of received stimulation affected learning rates. To this end, the sham group from each experiment was split into subjects who perceived the stimulation (‘Sham-felt’) and those who did not (‘Sham-no’). Interestingly, as shown in Figure 5A, in Experiment 1, the Sham-felt subjects (blue dashed line; *n* = 5) learned the rotation at a similar rate as those receiving 120 Hz TNS. On the contrary, the Sham-no subjects (blue dotted line; *n* = 16) initially performed in a manner similar to the 120 Hz subjects but later converged toward learning rates consistent with 60 Hz TNS, which was associated with the slowest learning rates. A similar behavior was observed in Experiment 2 (Figure 5B) where the Sham-felt subgroup (*n* = 8) demonstrated a similar learning rate compared to the active groups during the initial phase of learning but later converged toward the overall sham performance. In contrast, the Sham-no subgroup (*n* = 13) exhibited the slowest learning in the early phase of the adaptation but eventually converged to rates consistent with the overall sham levels by the end of the rotation block. The small numbers of subjects in each subgroup precluded statistical analyses of differences in learning rates between them. Nevertheless, these observations could have important implications for TNS applications and neuromodulation in general, as discussed below.

### 3.5 Other effects of TNS

In Experiments 1 and 2, 60/63 (~95%) and 61/63 (~97%) participants, respectively, completed the post-study survey. Table 3 summarizes these results. In both experiments, average levels of reported attention were higher than 7.8/10 across all groups, indicating that participants considered themselves to be engaged in the task. No statistically significant differences in reported attention levels among groups were found in either experiment (Kruskal-Wallis test, Exp 1: χ^2^(2) = 0.096, *p* < 0.953; Exp 2: χ^2^(2) = 0.253, *p* < 0.881). Nevertheless, we still explored potential correlations between reported attention levels and the two learning rates for each group. In Experiment 1, the 120 Hz group showed a moderately strong, positive correlation between attention level and the *α* coefficient (Pearson’s *r* = 0.48, *p* = 0.032). However, no other statistically significant correlations between reported attention level and learning rates were found in either experiment. In addition, we also explored potential correlations between reported attention levels and stimulation intensities. In Experiment 1, the 60 Hz group showed a moderate, positive correlation between reported attention level and tolerated current intensity (Pearson’s *r* = 0.53 *p* = 0.014). No other statistically significant correlations were found in either experiment.

**Table 3:** Post-study survey results for Experiments 1 and 2.

		120 Hz			60 Hz			Sham		
		Mean	SD	*n*	Mean	SD	*n*	Mean	SD	*n*
Attention Level	*Exp 1*	7.8	2.2	20	8	1.8	21	7.8	2.3	19
	*Exp 2*	8.1	1.8	19	8.5	1.3	21	8.2	1.8	21
Discomfort	*Exp 1*	4	–	1	3	1	3	5	4.2	2
	*Exp 2*	2.5	0.7	2	3.4	2.6	5	3.6	1	7
Headache	*Exp 1*	3	–	1	4.3	2.5	3	5	1.4	2
	*Exp 2*	2	–	1	4.6	1.8	5	5.3	3.5	3
Blurry vision	*Exp 1*	5.5	3.5	2	4	1.4	2	4	–	1
	*Exp 2*	2	–	1	5	–	1	–	–	0
Skin itching	*Exp 1*	3	1.4	2	6	–	1	–	–	0
	*Exp 2*	3	–	1	4	–	1	5	4.2	2
Dizziness	*Exp 1*	–	–	0	–	–	0	4.5	0.7	2
	*Exp 2*	7	–	1	3	0	2	6	–	1

Mean scores are reported on a scale of 1–10. For headaches, blurry vision, dizziness, itching and discomfort, 1 represents barely noticeable discomfort, and 10 represents unbearable pain. For the attention level, 1 represents not paying attention, and 10 indicates being engaged and anticipating stimuli.

In Experiment 1, 10% of all participants (6/60) reported mild discomfort (3.8/10 ± 2.2). When examined by groups, at least one participant per group reported discomfort. On the other hand, in Experiment 2, 23% of participants (14/61) reported mild discomfort (3.4/10 ± 1.7). Interestingly, the greatest number of participants reporting discomfort was in the sham group (*n* = 7, score = 3.6/10 ± 1). When consulted specifically for side effects such as headaches, blurry vision, dizziness, and skin itching at the electrode site, similar results were found. In Experiment 1, 15% or fewer participants in each group experienced a headache and 10% or fewer participants reported blurred vision. Only 10% of subjects in the sham group (but no other group) reported dizziness (*n* = 2, score = 4.5/10 ± 0.7) and skin itching occurred in fewer than 10% of participants and only in the stimulation groups. On the other hand, in Experiment 2, headaches were more commonly reported, with the 60 Hz group reporting five events, followed by the sham group with three, and the 120 Hz group with one. Only one participant in each active stimulation group reported blurred vision. Dizziness was reported in less than 10% of each group, similar to skin itching.

## 4 Discussion

Here we explored the effects of single-session TNS on visuomotor adaptation in healthy adults. These experiments were initiated with the long-term goal of evaluating the feasibility of using this neuromodulatory technique as an adjuvant to conventional neurorehabilitation of sensorimotor dysfunction. TNS was delivered online and offline in separate experiments, using stimulation parameters derived from clinical applications. We found evidence for both frequency- and timing-dependent effects of TNS on visuomotor adaptation. The results serve as a useful starting point for efforts aimed at developing optimal TNS parameters and protocols for augmenting conventional neurorehabilitation and enhancing human sensorimotor performance in industrial, athletic, military, and performing arts settings.

In Experiment 1, we assessed the effects of 60 and 120 Hz TNS on adaptation, hypothesizing that one or both frequencies would enhance learning rates during the adaptation block. However, that was not the case. Quantification of the learning rates associated with visuomotor adaptation showed that TNS at 60 Hz slowed the learning, an effect that was more pronounced in the early adaptation phase. More specifically, both the fast and slow learning rate coefficients were significantly smaller than those in both the 120 Hz and sham groups, suggesting that TNS at 60 Hz had a detrimental effect on learning mechanisms. On the other hand, although the 120 Hz group showed some evidence of enhanced learning relative to sham, changes in directional error were modest and were not statistically significant.

In Experiment 2, we assessed the same 120 and 60 Hz frequencies, but applied TNS during task performance (online). We expected to find more pronounced effects of TNS using this protocol, given the encouraging results observed using online transcranial electrical stimulation (Stagg et al., 2011; Pozdniakov et al., 2021; Martin et al., 2014). However, even though learning curves associated with the active stimulation groups showed evidence of slightly faster adaptation, learning rates analyses showed no statistically significant differences between the active groups rates and those of the sham group. Interestingly, when results from this experiment were compared *post hoc* with those observed in Experiment 1 (offline TNS), significantly faster learning rates were observed for the online 60 Hz protocol compared to offline. This indicates that the effects of TNS on visuomotor adaptation depend not only on the frequency of stimulation but also on timing relative to task performance, i.e., online vs. offline, at least for 60 Hz stimulation.

### 4.1 Potential neurophysiological mechanisms

Since only behavioral data were collected in these studies, the neurophysiological mechanisms underlying the observed effects are unknown at this time. The Aston-Jones model of locus coeruleus-norepinephrine (LC-NE) system function may provide a possible explanation for the observed frequency-dependent effects (Aston-Jones and Cohen, 2005). TNS effects are thought to be exerted via activation of the LC, with subsequent release of norepinephrine (NE) across the brain and resulting enhancement of synaptic plasticity (Mercante et al., 2017). Similar to the Yerkes-Dodson law that relates performance with arousal, the Aston-Jones model relates changes in task performance resulting from neuromodulation to changes in LC activity using an inverted U-shaped curve (Aston-Jones and Cohen, 2005). That is, poor performance is observed when either low or high tonic activity is present and more optimal performance occurs when moderate tonic and predominant phasic activity is present. In this scenario, the slower rates of learning observed with 60 Hz TNS relative to sham may have resulted from a shift in tonic LC firing, resulting in either very low or very high levels of tonic activity, both of which have been linked to poor performance. Along the same lines, learning rates for offline 120 Hz TNS, though not significantly different from sham rates in Exp. 1, were still among the highest observed and were significantly different from those obtained with offline 60 Hz TNS. Moreover, fast learning rates resulting from 120 Hz offline TNS were also moderately correlated with reported attention levels. This suggests that higher frequencies of stimulation might lead to more optimal levels of LC firing and enhanced arousal and learning. Effects of high frequency TNS on behavioral manifestations of motor learning are currently being explored in our laboratory (Arias and Buneo, 2022) but complimentary neurophysiological studies will be required to confirm this hypothesis.

The observed timing-dependent effects may reflect a difference in the timescales of neural mechanisms that are believed to support endogenous neuromodulation. For example, online effects of *transcranial* stimulation have been attributed to the modulation of cortical excitability through the alteration of neuronal membrane properties by weak electric currents (Yavari et al., 2018), a mechanism which acts on very short timescales. On the other hand, effects of transcutaneous cranial nerve stimulation are believed to occur via activation of brain stem nuclei, resulting in subsequent changes in cortical synaptic plasticity that happen on longer timescales. As a result, TNS modulation might be expected to be more amenable to offline rather than online stimulation, as it would allow more time for such changes to take hold. However, the opposite was observed here, at least with 60 Hz TNS, i.e., faster learning was observed with the online protocol compared to the offline protocol. It is possible then that TNS effects are mediated by both transcranial and transcutaneous mechanisms. Transcranial mechanisms might be invoked due to the location of the electrodes, which would allow some current to reach frontal cortical areas, a scenario supported by current flow models (Schutter et al., 2019). To determine whether transcranial, transcutaneous or both mechanisms underlie TNS effects, future studies might consider blocking the trigeminal nerve using skin anesthetics to eliminate or reduce contributions from peripheral cutaneous receptors. This general approach was recently used to probe the source of behavioral effects when tDCS was applied to the posterior cervical region. Residual behavioral effects of stimulation were observed even after blocking the occipital nerve (Vanneste et al., 2020), suggesting that some effects of stimulation in this region result via transcranial mechanisms, which could be same for TNS.

### 4.2 Sham groups performance

In both experiments, a subset of participants in the sham group (24% in Exp. 1 and 38% in Exp. 2) reported perceiving the stimulation even though no current was applied to them. Interestingly, these participants learned the rotation at a rate similar to the best performers of each experiment. Thus, in Experiment 1, the sham-felt subgroup tracked the learning behavior of the 120 Hz group while in Experiment 2, the sham-felt tracked the 120 and 60 Hz groups early during the adaptation and then diverged in the late stage, ending up with higher directional error. On the other hand, participants who did not report perceiving stimulation (sham-no) generally demonstrated the slowest learning rates. Placebo effects are thought to result from a combination of factors such as the environment, expectations about the treatment, as well as physiological effects. Environmental factors appear to play a key role in transcranial neuromodulation studies, where strong placebo effects have been reported (Burke et al., 2019). The presence of equipment sounds generated by this equipment, the placement of electrodes on the head, and interactions with the experimenter, among other factors, can potentially provide cues to participants about the treatment, contributing to placebo effects. In our protocol, subjects in the sham groups used the same electrode placement as the active groups, the stimulator made the same clicking sound every time it was supposed to be active, and the experimenter mimicked the same protocol used for the active groups to look for submaximal tolerable thresholds, but no current was delivered. Nevertheless, some participants still performed in a manner suggestive of a placebo effect. Furthermore, the expectation of receiving a treatment, i.e., stimulation or a drug, has also been shown to be a relevant factor in placebo/sham-controlled studies and may have contributed to the apparent placebo effects observed here. Although we did not probe participants’ expectations in our survey, those who perceived the stimulation might have had a different expectation from those who did not perceive it. That is, not feeling the stimulation might have lowered expectations in these participants, potentially affecting their performance. In contrast, feeling the stimulation might have affected the performance of those participants positively.

We decided on a passive sham approach based on a review of the tDCS literature, which indicated that an active sham, i.e., the delivery of current for a small period of time in order to evoke cutaneous responses, can still produce neural effects, even at low intensities of stimulation (Nikolin et al., 2018). Thus, our passive sham approach also served as a non-stimulation control. However, although a small percentage of participants in the sham group perceived stimulation despite no current being delivered, it is important to acknowledge that the passive sham approach, combined with the cutaneous sensations evoked by TNS, makes it nearly impossible to fully blind the participants, which represents a limitation of the present study.

### 4.3 Side effects

Ten percent (10%) of participants in Experiment 1 reported experiencing some discomfort during the experiment, while 23% reported discomfort in Experiment 2. This is surprising given that participants were instructed to select a comfortable intensity and were told that they could ask to reduce or stop the stimulation at any time. Additionally, subjects in the sham group also reported experiencing discomfort, even though they did not receive any current. Importantly, our questionnaire did not probe the source of the reported discomfort, thus it is possible that this perception was unrelated to the stimulation. For instance, several participants across all groups complained about arm fatigue, which could have been source of this discomfort due to the highly repetitive nature of the reaching task that was used.

Participants were also asked specifically about whether they experienced headaches, blurry vision, skin itching, and dizziness in both experiments. In Experiment 2, headaches were reported by the largest number of participants, with five (23.8%) in the 60 Hz group, followed by three (14.3%) in the sham group and only one in the 120 Hz group (5.3%). These findings align with the results of Experiment 1, where three (14.3%) instances were reported in the 60 Hz group, two (10.5%) in the sham group and one in the 120 Hz group (5%). Although headaches and skin itching have been associated with TNS in other studies (DeGiorgio et al., 2013; McGough et al., 2019), in these experiments it is difficult to attribute them exclusively to the stimulation because subjects in the sham group also reported experiencing them, even though no current was applied. This suggests that some of these headaches may have resulted from experimental factors other than the stimulation. For example, prolonged exposure to VR environments is known to contribute to cybersickness, which manifests as headaches and eyestrain, among other side effects (Martirosov et al., 2022). Given the relatively long duration of our experiments (2 h), it is possible that our VR environment, and not the TNS, was the root cause of some or most reports of headache. In support of this idea, it is noteworthy that one participant discontinued the experiment due to eyestrain.

### 4.4 Stimulation parameters and protocol

In these experiments, several stimulation parameters were tested, which to our judgment provided reasonable starting points to assess the feasibility of the effects of TNS on motor learning. However, these parameters are clearly a minimal sample of the overall stimulation parameter space. For example, we addressed the effects of a single session of approximately 20 min of cycled stimulation (30 s ON/OFF), which, as described above, produced modest effects. The selected stimulation duration for these studies corresponds to the daily dose of TNS for migraine (Schoenen et al., 2013), which has also been used in healthy adults to assess the neural effects of TNS (Ginatempo et al., 2018a; Mercante et al., 2015). However, in previous studies, therapeutic effects of TNS have been shown to require weeks or months of treatment (McGough et al., 2019), with an even longer daily dose. Therefore, increasing the session length and/or holding multiple sessions across several days might enhance the effects of TNS on motor learning. Moreover, we utilized cycled stimulation because it has been commonly used clinically to address the symptoms of ADHD and DRE (DeGiorgio et al., 2013; McGough et al., 2019). However, continuous TNS has been shown to have beneficial effects on migraine (Schoenen et al., 2013), which warrants further investigation into this mode of application in studies of TNS efficacy. Finally, future work should consider pairing the stimulation with movements, which have shown to be effective for rehabilitation using VNS (Dawson et al., 2021).

In both experiments, the stimulation intensity was determined by each participant’s self-selected submaximal tolerable threshold, which offered participants more control over the dose they received. Although we found that mean currents did not differ significantly across groups in either experiment, current levels within each group were highly variable, which might have contributed to the modest differences among groups reported here. Given that learning rates within each group were also somewhat variable within each group, we explored potential associations between learning rates and intensities, but statistically significant correlations were only found for the 60 Hz group in Experiment 1. An alternative approach would have involved using a fixed current for all subjects, similar to the approach used in tDCS studies, where a fixed intensity of 2 mA is typically employed. Determining whether fixed vs. self-selected TNS intensities yields more favorable results with regard to the effects on motor learning will require further investigation.

### 4.5 Behavioral task

Here, a visuomotor rotation task was used to study motor adaptation, a form of motor learning where previously learned movements are adapted following a transient perturbation in the environment. This task was chosen because it has been extensively studied both as a means to understand motor learning in general and also as a means to quantify the effects of neuromodulation on motor learning (Galea et al., 2011; Herzfeld et al., 2014). Despite the evidence provided here suggesting that TNS affects motor learning behavior, the magnitudes of these effects were modest. Results obtained using offline 120 Hz stimulation were not statistically different from sham. This suggests that TNS does not improve the rate of visuomotor learning in healthy young adults. However, recent studies using older adults suggest that the degree of visuomotor adaptation is reduced and more variable in this population (Wolpe et al., 2020). Thus, future studies could focus on assessing the effects of TNS on visuomotor performance using the same paradigm but with an older population. This way, a better understanding of the potential for TNS to enhance neuroplasticity and motor learning in ‘age-matched’ stroke survivors could be obtained.

It has been shown that some participants with a high level of motor expertise such as athletes (Leukel et al., 2015) and minimally invasive surgeons (Hewitson et al., 2020) learn visuomotor perturbations at a faster pace than non-expert controls. In addition, people with specific genetic profiles, such as a polymorphism of the brain-derived neurotrophic factor (BDNF) gene, have been shown to exhibit different learning rates than control subjects (Joundi et al., 2012). Additional insights into the effects of TNS on visuomotor learning might be obtained by examining such populations. Other tasks falling under the motor learning category such as the serial reaction time task (Robertson, 2007) could also potentially be used to assess the effects of TNS on learning. Lastly, more clinically relevant tasks should be explored to evaluate the potential efficacy of TNS on sensorimotor dysfunction in both older adults and neurologically impaired individuals, which could also potentially reveal larger effect sizes.

## Data Availability

The original contributions presented in the study are included in the article/Supplementary material, further inquiries can be directed to the corresponding author.

## Footnotes

^1^https://www.sealedenvelope.com/
