# Supplementary material for: Effects of online and offline trigeminal nerve stimulation on visuomotor learning
Source: Front Hum Neurosci. 2024 Oct 17;18:1436365. doi: 10.3389/fnhum.2024.1436365 (PMC11526447; doi:10.3389/fnhum.2024.1436365)
Supplement: Supplementary file 2 [file Table_1.docx]

**Table 1. Subject genders and ages in Experiment 1.**

To determine whether there were any significant differences among the subgroups in terms of gender in Experiment 1, a Chi Square test of independence was performed. This test showed no statistically significant differences among the groups (χ^2^(2)=2.4133, p=0.2992) indicating that subgroup and gender were independent.

**Table 2. Subject genders and ages in Experiment 2.**

*For Experiment 2, the genders were more unbalanced. As a result, Fisher's Exact Test for Count Data was used to determine whether there were any significant differences among the subgroups in terms of gender. This also showed no statistically significant differences among groups (p=0.3498).*

**Table 3. Average RTs and PVs across subjects each block/phase of Experiment 1.**

**Table 4. Average RTs and PVs across subjects each block/phase of Experiment 2.**
